# Tuberculin

**Published:** 1907-09

**Authors:** 


### TUBERCULIN:

E. L. Trudeau states that in tuberculosis, the more chronic type of the disease, the better adapted to tuberculin treatment the case seemed to him, and that most cases of a chronic type, whether incipient or of long standing and advanced, provided the nutrition is good and no serious complications exist, will derive more or less benefit from tuberculin immunization. The benefit derived may be from mere temporary improvement in all the symptoms, to apparent restoration to health, with disappearance of bacilli often lasting for years. All acute cases, early cases in which the onset is acute, or advanced cases with active symptoms, should be treated by the rest and open air method only. If they progress to cure by this method it is unnecessary to use tuberculin, and indeed he has formed the impression that it is better not to use it during the periods of activity or at least until a partial arrest in the activity of the infection manifests itself When the physician becomes satisfied that the patient is no longer making improvement while treated by the open air method, and that the case has become one of chronic phthisis, if the general nutrition is good, tuberculin injections offer a prospect of further improvement. All acute cases, cases with long continued increasing fever, cases with very extensive lesions and that are very feeble and badly nourished, cases with intestinal and other complications and patients who show pronounced and uncontrollable intolerance to tuberculin, are manifestly unsuited for treatment. That tuberculin is not the vaunted and long-looked-for specific, it was first thought to be, has been amply demonstrated by the bitter experience of the past. We have learned the dangers of tuberculin treatment and its evident limitations. He is inclined to think that the production of tuberculin immunity by the mild clinical method will favorably influence chronic tuberculosis, prolonging life and in many cases will abort a commencing infection or extinguish the smouldering fires of a chronic infection.—American Jour. Med. Sciences, June 1907.

